# Validity of OSDI-6 questionnaire in a Chinese adult population

**DOI:** 10.1038/s41598-024-64953-1

**Published:** 2024-06-21

**Authors:** Guanghao Qin, Salissou Moutari, Jiayan Chen, Ling Xu, Wei He, Xingru He, Emmanuel Eric Pazo, Sile Yu

**Affiliations:** 1 He Eye Specialist Hospital, Shenyang, China; 2 School of Mathematics and Physics, Queens University Belfast, Belfast, UK; 3 School of Public Health, He University, Shenyang, 110034 China

**Keywords:** Dry eye disease, OSDI, OSDI-6, Rasch analysis, Diseases, Medical research

## Abstract

This study aimed to evaluate the validity of the Chinese translation version of OSDI-6 (C-OSDI-6) using a virtual set-up questionnaire for dry eye disease. A total of 270 participants (136 males, 50.4% and 134 females, 49.6%) with a mean age of 28.22 ± 9.01 years were assessed, diagnosed under the criteria put forth by Dry Eye Workshop completed the Chinese translated version of the OSDI-12 questionnaire (C-OSDI-12). Validity and psychometric properties were analyzed using the study data on the selected items (a new approach called virtual validation). The six items were extracted from the C-OSDI-12 as suggested by the authors of OSDI-6 and compared. The total scores of C-OSDI-12 and C-OSDI-6 were 30.27 ± 13.19 and 6.95 ± 3.53, respectively. Significant reliability was found between the total C-OSDI-6 score and the total C-OSDI-12 score (r = 0.865, p < 0.001). Infits and outfits of the C-OSDI-6 were between 1.26 and 0.78.The C-OSDI-6 proved valid and psychometrically responsive in Chinese adult dry eye participants. The findings of this virtual validation study need to be confirmed in a longitudinal validation study on real-world use.

## Introduction

Dry eye disease (DED) is stated to have a worldwide incidence of between 5 and 50% and is prevalent in China^1–3^. It is reported to be one of the most common reasons for patients visiting their eye care practitioner^4^. While DED was previously defined as ocular dryness caused by a decrease in the aqueous component of the tear film, the current TFOS DEWS definition states that "Dry eye is a multifactorial disease of the tears and ocular surface that manifests as discomfort, visual disturbance, and tear film instability, with the potential for ocular surface damage"^5^. Screening for dry eye symptoms and quantifying dry eye symptoms aids in assessment, diagnosis, and treatment^6^. Patient-reported outcome (PRO) instruments such as the Ocular Surface Disease Index (OSDI-12) are utilized for assessing patients' subjective experiences and functional abilities^7^. The OSDI-12 comprises of 12-item and is often used in conjunction with other tests in clinical settings to assess individuals with DED. Created in 1997, the original OSDI consisted of 12 items and was designed to evaluate subjective dry eye symptoms and the consequences of DED on vision-related activities of daily life over the preceding week^8^. The 12 items are grouped into three subscales: ocular symptoms (three items), vision-related functions (six items), and environmental triggers (three items). While the original version of the OSDI-12 questionnaire was created in English and was later translated and validated into Arabic^9^, Bahasa^10^, Chinese^11^, Farsi^12^, Filipino^13^, Japanese^14^, Lithuanian^15^, Portuguese^16^, and Spanish^17^, it should be used carefully since the cutoff values vary among various languages (27.2 points for the Chinese version and 36.3 points for the Japanese version)^14,18^. Recent research using Rasch analysis found that separating answer items into four categories by merging "half of the time" and "most of the time" might lead to higher thresholds and better intervals in each category^19^. The OSDI-12 total score is calculated using the formula: [(sum of scores for all questions answered) × 100]/ [(total number of questions answered) × 4]. Ocular Surface Disease Index-6 (OSDI-6) is a derivative and shortened version of the original OSDI-12 and can potentially improve efficiency in a busy clinical setting. According to Pult et al.^20^, OSDI-6 was found to have strong repeatability and can be used as an alternative to OSDI-12 in a clinical setting. The six items of OSDI-6 are all derived from the twelve items of OSDI-12 and can be calculated by the new formula (OSDI-6 total score = (Item 1 + Item 4 + Item 7 + Item 9 + Item 10 + Item 11). The increasing demand for eye care and outpatient clinicians in China and around the world^21,22^ suggests that a shortened DED questionnaire, such as the OSDI-6, can improve efficiency in the clinical workflow. However, the viewpoint of shortening a lengthy questionnaire to increase the rate of response has not yet been entirely resolved. While the original OSDI-12 has been tested in various countries, including China^11,18,23^, the validity and psychometric properties of the Chinese version of OSDI-6 (C-OSDI-6) have not been tested. Furthermore, it has been found that the results of health-related surveys might be culturally biased or neutral^24,25^. Since testing clinical questionnaires' validity is essential^26^. This study used Rasch analysis to evaluate the validity of the Chinese translation version of the OSDI-6 using a virtual set-up questionnaire.

## Methods and subjects

### Ethical approval

The research conducted at He Eye Specialist Hospital was evaluated by the Institutional Review Board (IRB) in accordance with the principles outlined in the Declaration of Helsinki. The research was approved under the reference number IRB(2023)K032.01 and registered with the Registry of Clinical Trials under the identifier NCT06158906. All participants completed informed consent forms after being fully apprised of the study's risks and consequences. Participants' data were gathered from December 2023 to February 2024. Clinically diagnosed DED participants answered the Chinese web version of the C-OSDI-12 questionnaire, which was previously translated and validated Chinese version of OSDI (Allergan Inc, Irvine, USA). The questionnaire's 12 items can be tabulated for a score ranging from 0 (no symptoms) to 100 (severe symptoms).

### Clinical assessment

For this prospective study, 310 consecutive consenting people were recruited from the DED clinic. Diagnostic criteria for DED were as follows: At least one of the following six symptoms is present: dryness, burning, sandiness, weariness, eye discomfort, and/or poor vision, all accompanied by a non-invasive tear breakup time (NITBUT) of fewer than ten seconds or a corneal fluorescein staining (CFS) score. The inclusion criteria were as follows: (1) full legal age, (2) Both eyes meet DED diagnosis, (3) compliance with research instructions, (4) independent reading and understanding of the C-OSDI-12 questionnaire, (5) completion of the whole study process, and production of signed consent, (6) has not received any treatment for DED. Exclusion criteria included: (1) inability to provide informed consent and (2) participation in other studies (burden of participation), (3) prior ocular surgery or trauma, (4) acute inflammation, (5) blepharal or periorbital skin disease or allergies in the preceding month, (6) rheumatic immune systemic diseases, (7) pregnancy, and 8) breastfeeding.

### Experimental design

**Figure 1:**
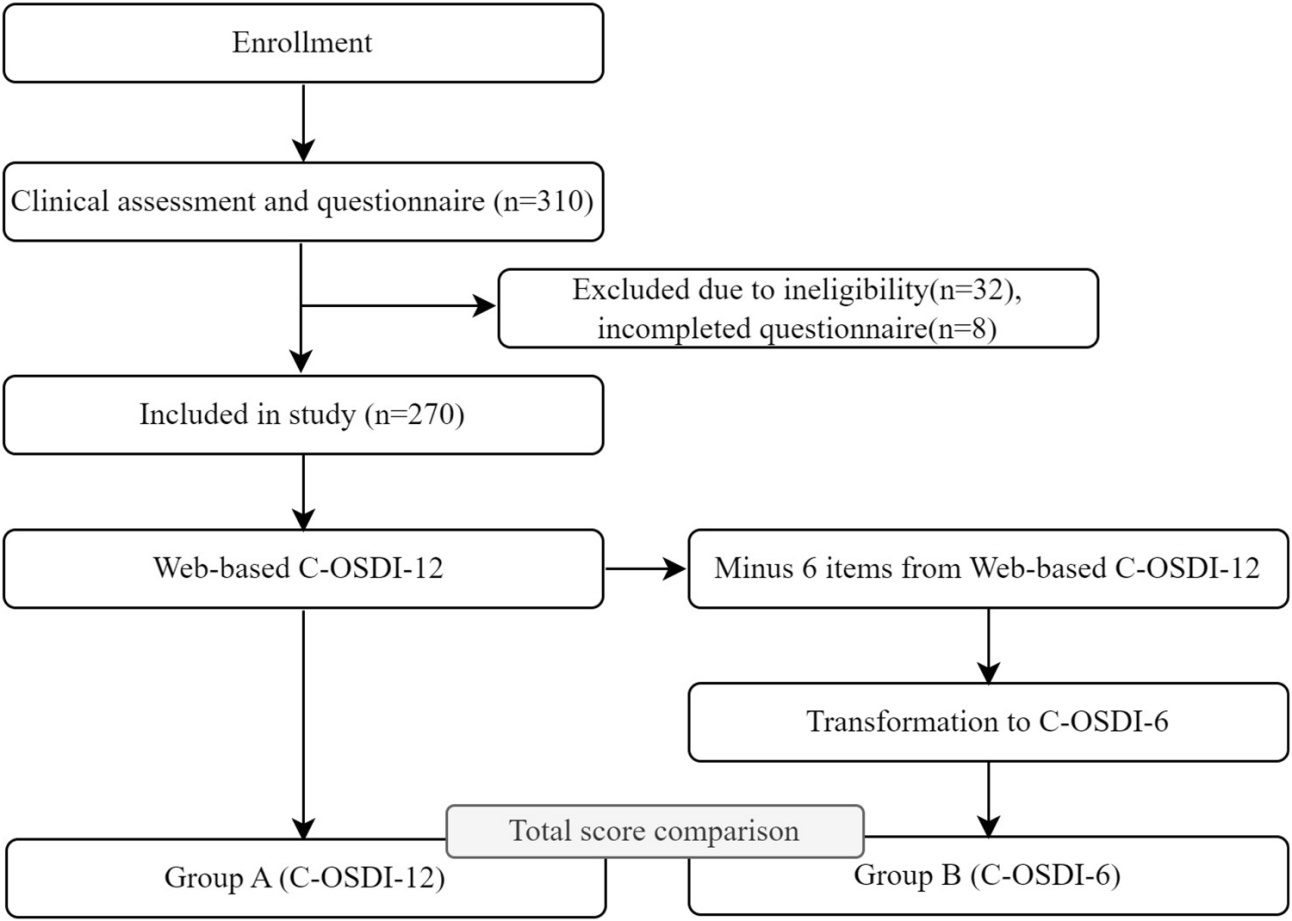
Study flow diagram.

Three hundred and ten patients between 18 and 62 years were evaluated for eligibility, and 270 patients were finally included in this study. Participants were assessed for eligibility at their first clinic visit, and those who met the criteria were invited to engage in the study. Participants were asked to complete questionnaires during their first-time hospital visit under the supervision of qualified doctors (Fig. 1).

### Questionnaire

**Figure 2:**
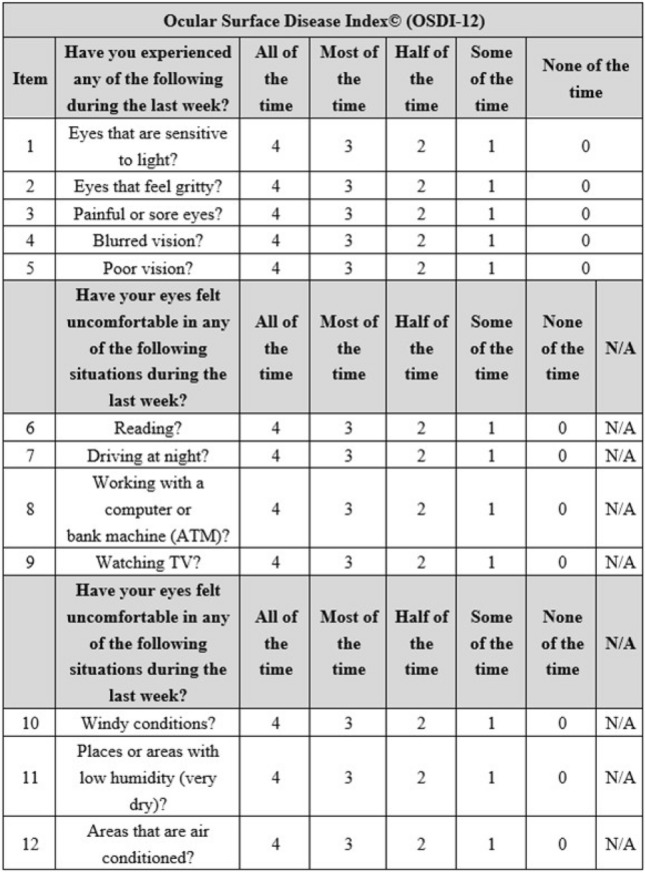
Ocular Surface Disease Index-12 (OSDI-12) Questionnaire.

**Figure 3:**
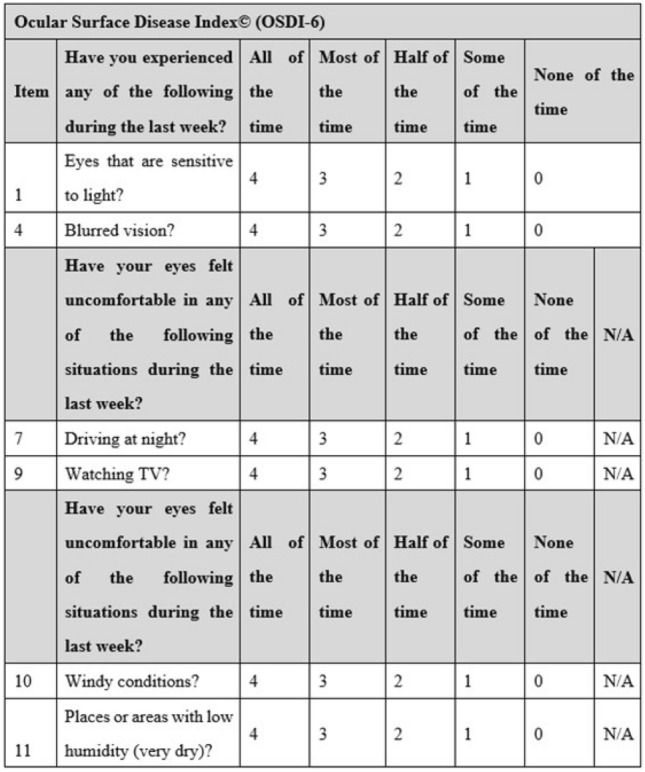
Ocular Surface Disease Index-6 (OSDI-6) Questionnaire.

The so-called theoretical validation, as proposed by Blome et al.^27^ and successfully conducted by Augustin M et al.^28^ for the development and validation of a short version of the Freiburg Life Quality Assessment for chronic venous disease (FLQA-VS-10) questionnaire, was carried out. The current study used the item score data obtained from C-OSDI-12 to derive data for C-OSDI-6 since the original C-OSDI-12 questionnaire shares six common items with C-OSDI-6. Thus, in the virtual validation, the total C-OSDI-12 (Fig. 2) score was computed as: [(Item 1 + Item 2 + Item 3 + Item 4 + Item 5 + Item 6 + Item 7 + Item 8 + Item 9 + Item 10 + Item 11 + Item 12) × 100]/[(total number of questions answered) × 4] (standard recommendation) while the total C-OSDI-6 score was derived from the OSDI-12 data and computed as : Item 1 + Item 4 + Item 7 + Item 9 + Item 10 + Item 11(standard recommendation) (Fig. 3).

### DED assessment

The following tests were performed on both eyes. NITBUT was determined using the Keratograph 5 M (Oculus, Germany) three times consecutively. The final analysis was conducted using the median value. Interferometry of the tear film lipid layer (TFLL): DR-1 (Kowa, Nagoya, Japan) was used to rate the TFLL quality on a scale of 1 to 5 (1: gray uniform), 2: gray non-uniform), 3: few colors non-uniform, grade 4: many colors non-uniform, grade 5: partly exposed corneal surface), using the Hosaka et al. severity grading method^29^. CFS: A preservative-free solution containing 1% sodium fluorescein was instilled in the conjunctival sac. The cornea was graded using the Efron method and assigned a score between 0(normal), 1(trace), 2(mild), 3(moderate), and 4(severe)^30^. The Keratograph 5 M was used to measure conjunctival hyperemia (CH) (Oculus, Germany)^31^.

### Statistical analysis

All statistical analyses for this investigation were completed using SPSS (IBM, version 25). Eight incomplete questionnaires were not included in the final analysis (Fig. 1). The descriptive sociodemographic parameters of patients were generated by studying the complete data's frequency distribution. We examined the convergent validity using Spearman's correlation of the total scores of C-OSDI-12 and C-OSDI-6. Shapiro–Wilk test indicated that the paired samples were not normally distributed. In this investigation, P < 0.05 (2-tailed) was regarded statistically significant difference (i.e., the level of significance α = 0.05). The psychometric features of C-OSDI-12 and C-OSDI-6 questionnaire answers were examined employing Rasch analysis^19^. Further details and background to Rasch analysis in ophthalmic research can be found in McNeely et al.^19^. The sample size for this crossover design comparisons research was estimated using the equation: (1-ρ)/2, where ρ is an estimate of the predicted correlation between the two modalities of administration^32^.

### Assessment of the Rasch analysis

The validation of the psychometric quality of questionnaires using Rasch analysis is commonly based on goodness of fit measures derived from the standardized residuals. The residuals represent the part of the information within the observed data not captured by the model. Large values of the residuals meant the model failed to describe significant details within the observed data.

### Misfit statistics

The goodness of fit measures used in the context of Rasch analysis is known as infit and outfit test statistics and are also referred to as misfit statistics. The outfit statistic is a measure sensitive to unexpected observations by persons on items, i.e., it is susceptible to outliers within the observed data. On the other hand, the infit statistic is a measure sensitive to unexpected patterns of observations by persons on items targeted for them, i.e., it is sensitive to the patterns within the observed data.

The estimated values commonly used to analyze the outfit and infit statistics are the mean-squares fit statistics, denoted Outfit MNSQ and Intfit MNSQ. These values represent the level of randomness with the observed data and are expected to be clustered around the value 1. When the values of MNSQ are low compared to 1, then the model is overfitted. On the other hand, when the values of MNSQ are high compared to 1, the model provided a poor representation of the observed data. A general guideline regarding the cutoff values for the Outfit MNSQ and the Intfit MNSQ is that for a good fit of the observed data to the model, these values should be within the range of 0.7 to 1.30 logit^33,34^. Therefore, any item with an Outfit MNSQ or an Intfit MNSQ value outside this range can be removed from the analysis.

### Principal component analysis (PCA)

PCA is another method employed to evaluate the standardized residuals in Rasch analysis. An eigenvalue of the first principal component greater than 1.4 suggested violating the unidimensionality assumption, which is critical for the validity of the Rasch model.

### Normality test

The standardized residuals from the Rasch analysis can be assessed using the test normality. The test evaluated how well the distribution of the Rasch residuals fitted to a standard normal distribution, i.e., how symmetric and centered around 0 the distribution. At the same time, the peak is neither too high nor too low. Such a feature confirms the critical unidimensionality assumption of the Rasch model, hence its psychometric quality.

### Ethics approval and consent to participate

This study was conducted following the ethical standards of the declaration of Helsinki. The study was carried out following the He Eye Specialist Hospital, Institutional Review Board regulations and approval. Documented informed consent was obtained from all participants in this study. In the present study, all components with any individually identifiable information have been removed from the dataset, which classifies it can be used as non-human subjects' research.

## Results

Table 1 summarizes the demographic and clinical data of study participants. The final analysis comprised 270 dry eye patients who completed the C-OSDI-12 after signing an informed written consent form and were categorized as DED according to the Dry Eye Workshop's (DEWS) criteria. With an average age of 28.22 ± 9.01 years, the research included 136 (50.4%) male and 134 (49.6%) female participants, and the mean IOP was 14.09 ± 1.37 mmHg.

**Table 1:** Demographic and clinical information on study participants (n = 270).

Variable	
Age (years)	
Mean ± SD	28.22 ± 9.01
IQR	21 to 32.25
Gender, n, %	
Male	136 (50.4%)
Female	134 (49.6%)
Education level, n, %	
Primary	0 (0%)
Secondary	3 (1.1%)
Tertiary	267 (98.9%)
Vision correction, n, %	
Spectacles	109 (40.4%)
Contact lens	87 (32.2%)
Refractive surgery	31 (11.5%)
None	43 (15.9%)
NITBUT (seconds)	
Mean ± SD	6.37 ± 1.96
IQR	4.92—7.97
Corneal staining (Efron scale: 0–4)	
Mean ± SD	1.12 ± 0.7
IQR	1.00—2.00
Tear lipid layer (DR-1 scale: 0–4)	
Mean ± SD	2.63 ± 0.65
IQR	2.00—3.00
Conjunctival hyperemia (Oculus scale)	
Mean ± SD	1.07 ± 0.37
IQR	0.80—1.30
IOP (mmHg)	
Mean ± SD	14.09 ± 1.37
IQR	14—15

*SD* Standard deviation, *IQR* Interquartile range, *NITBUT* Non-invasive tear breakup time, *IOP* Intraocular pressure.

**Figure 4:**
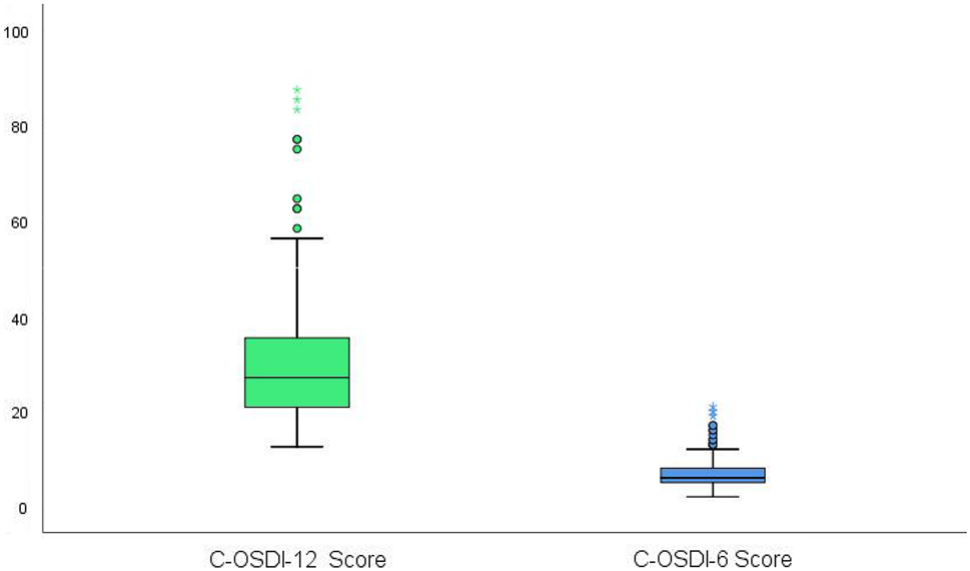
Boxplot distribution of C-OSDI and C-OSDI-6 (Chinese Ocular Surface Disease Index-6) total scores.

**Figure 5:**
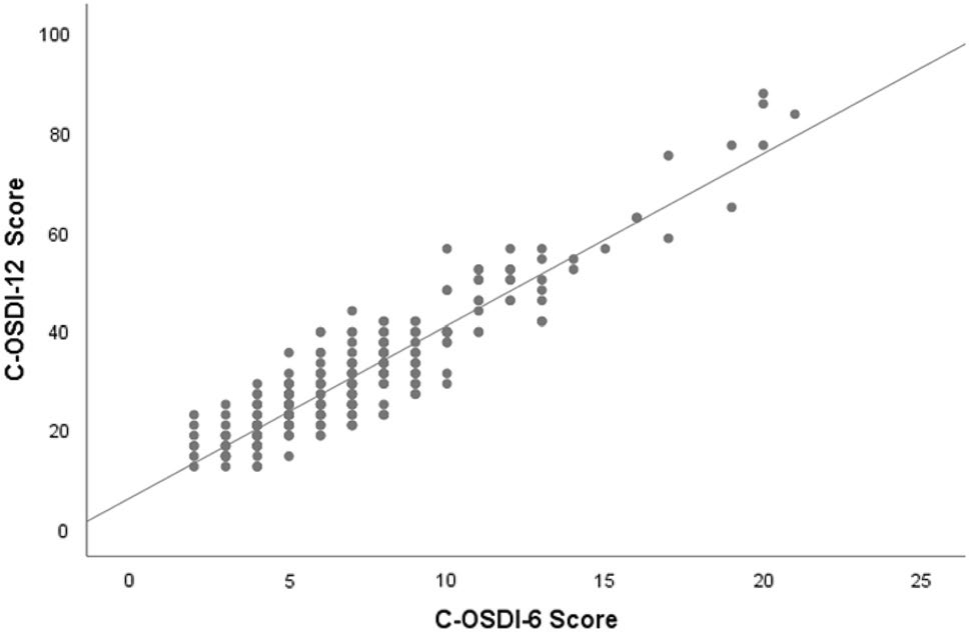
Spearman’s correlation between C-OSDI-12 and C-OSDI-6 (Chinese Ocular Surface Disease Index-6) total scores.

The total mean questionnaire scores using the C-OSDI-12 were 30.27 ± 13.19, and the derived total mean score from C-OSDI-6 was 6.95 ± 3.53. The scores for each item are shown in Table 2. The distribution of total scores for C-OSDI-12 and C-OSDI-6 are illustrated as box plots (Fig. 4). There is a positive correlation between the total scores of C-OSDI-12 and C-OSDI-6 (r = 0.865, p < 0.001) (Fig. 5).

**Table 2:** Single items, subscale, and total score.

Item	Mean ± SD	IQR
Item 1	1.11 ± 0.98	0.75–1.00
Item 2	1.17 ± 0.91	1.00–1.00
Item 3	1.18 ± 0.90	1.00–1.00
Item 4	1.31 ± 0.96	1.00–2.00
Item 5	1.30 ± 1.11	1.00–2.00
Item 6	1.22 ± 1.09	0.00–2.00
Item 7	0.96 ± 0.97	0.00–1.00
Item 8	1.35 ± 0.97	1.00–2.00
Item 9	1.20 ± 0.93	1.00–2.00
Item 10	1.20 ± 0.89	1.00–2.00
Item 11	1.18 ± 0.85	1.00–2.00
Item 12	1.36 ± 0.86	1.00–2.00
Total C-OSDI-12 score	30.27 ± 13.19	20.83–35.94
Total C-OSDI-6 score	6.95 ± 3.53	4.75–8.00

Rasch analysis was used to investigate the validity of the C-OSDI-6 questionnaire using only the 6 items as suggested by Pult and Wolffsohn et al.^20^. The infits MSQ and outfit of the C-OSDI-6 (Table 3) are within the range 0.70- 1.30 logit, as suggested in the literature^33,34^ item 5 appears to be misfitted in C-OSDI-12 (Table 4), with both infit and outfit values greater than 1.30. Furthermore, the principal component analyses of the standardized residuals from the Rasch analyses of C-OSDI-6 gave an eigenvalue of the first principal component of 0.806, which indicates the unidimensionality feature of the C-OSDI-6. The first eigenvalue of the first principal component has a value of 1.325 for C-OSDI-12. Since the eigenvalue of the first principal component when using C-OSDI-6 is less than the cutoff value of 1.40, suggested in the literature. The reduction of scale from 12 to 6 doesn't affect the psychometric properties of the C-OSDI questionnaire, namely the critical unidimensionality assumption of the Rasch model, in a clinical population.

**Table 3:** Infits and outfits statistics for C-OSDI-6.

Items	Infits	Outfits
Q1	1.1	1.03
Q4	0.85	0.83
Q7	1.29	1.29
Q9	1.05	1.09
Q10	0.85	0.83
Q11	0.93	0.94

**Table 4:** Infits and outfits statistics for C-OSDI-12.

Items	Infits	Outfits
Item 1	1.02	1.03
Item 2	0.78	0.75
Item 3	0.93	0.94
Item 4	0.78	0.78
Item 5	1.48	1.44
Item 6	1.18	1.22
Item 7	1.24	1.23
Item 8	1.03	1.1
Item 9	0.92	0.98
Item 10	0.88	0.94
Item 11	0.86	0.91
Item 12	0.97	1.05

**Figure 6:**
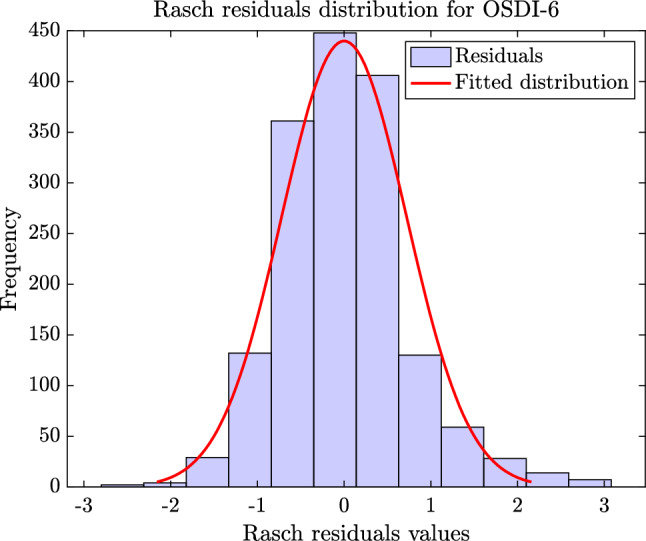
Distribution of residuals from the Rasch analysis of OSDI-6.

**Figure 7:**
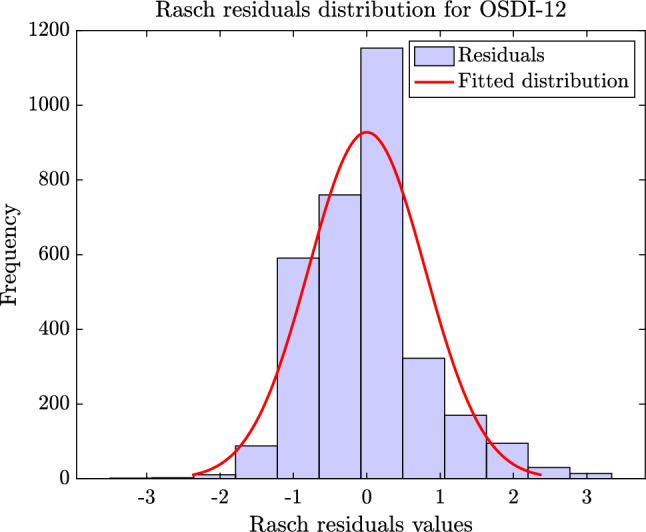
Distribution of residuals from the Rasch analysis of C-OSDI-12.

Figures 6 and 7 present the fitting of the standardized residuals from the Rasch analysis of C-OSDI-6 and C-OSDI-12, respectively, to the normal distribution. From these figures, the distribution of the Rasch residuals for C-OSDI-6 (Fig. 6) is clearly more symmetric and centered around 0 compared to the distribution of the Rasch residuals for C-OSDI-12 (Fig. 7). Furthermore, the results from the Shapiro–Wilk normality test suggested that there isn't enough evidence to reject the hypothesis that the C-OSDI-6 standardized residuals come from the normal distribution at the significant level of 5% (p-value = 0.066). In contrast, there is enough evidence to reject the hypothesis that the C-OSDI-12 standardized residuals come from the normal distribution at the significant level of 5% (p-value = 0.043). These results highlighted an improved psychometric feature of C-OSDI-6 compared to C-OSDI-12.

**Figure 8:**
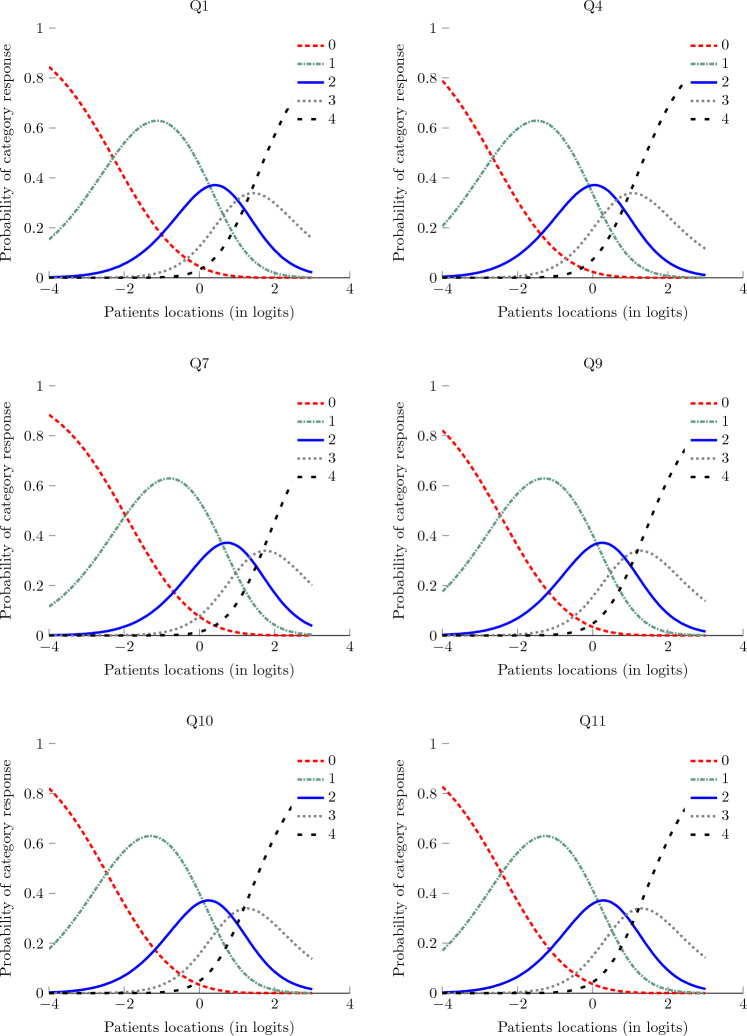
Item characteristic curves for the 6 items of OSDI-6.

Figure 8 presents the item's characteristic curves (ICC) of the OSDI-6 questionnaire. These curves represent the relevance of the response to items using the different ratings. Relevant ratings in the response will have their curves above for a reasonable range of the patients' locations (in logit). The analysis of the ICC in Fig. 8 suggested that for C-OSDI-6, the ratings 2 and 3 can be collapsed, and thus 4 rating scales instead of 5 could be enough to capture the underlying latent trait. The item characteristic curve for rating 3 is rarely above the characteristic curves of the two adjacent ratings, namely rating 2 and 4. Thus the suggestion to collapse ratings 2 and 3.

## Discussion

This study initially assessed the validity and psychometric reliability of the C-OSDI-6 in measuring subjective symptoms associated with DED. In comparison to the original twelve items of OSDI-12, C-OSDI-6 removes items 2 (Eyes that feel gritty?), 3 (Painful or sore eyes?), 5 (Poor vision?), 6 (Difficulty reading), 8 (Difficulty working with a computer or bank machine), and 12 (Uncomfortable in areas that are air-conditioned?), leaving behind items 1(Eyes that are sensitive to light?), 4 (Blurred vision?), 7 (Driving at night?), 9 (Watching TV?), 10 (Windy conditions?), and 11 (Places or areas with low humidity). The findings of the Spearman analysis suggest that C-OSDI-6 has a high level of reliability. Additionally, according to Rasch analysis, reducing the scale from 12 to 6 items has no effect on the C-OSDI-6 questionnaire's psychometric qualities in a clinical population.

Ophthalmic clinicians in China are frequently overworked with high volumes of patients^22^. Research suggests that fatigue was common among clinicians working in ophthalmology, especially during the COVID-19 pandemic^35,36^. Healthcare profession is known for its high stress, fatigue, and burnout^36^, which can consequently lead to significant medical blunders^37^. Moreover, excessive workloads might jeopardize physicians' health and the quality of their patient care^38^ Therefore, reducing the items of the original OSDI-12 questionnaire without compromising on its accuracy is a much-appreciated help in a fast-paced ophthalmic center^39^. Simultaneously, the patient experiences relief from the abbreviated questionnaire during their visit to the ophthalmic clinic.

The high prevalence of DED in China^21^ coupled with the altered day-to-day lifestyle due global COVID-19 pandemic, dry eye symptoms have been reported to have increased as a result of prolonged screen time and face mask usage^40^. Conversely, various isolation measures make it difficult for DED patients to visit eye clinics regularly. Therefore, using a brief web-based C-OSDI-6 to assess symptoms of DED allows for a convenient self-assessment tool. Additionally, it enables patients to make timely lifestyle modifications to improve their symptoms^41^.

Despite its satisfactory measurement properties, the length of a PRO instrument or a questionnaire may severely limit its application in practical settings. Hence, there is a significant interest in producing shorter PRO instruments and reducing the existing ones. Two main approaches are commonly used to assess the quality of PRO instruments: classical test theory (CTT) and item response theory (IRT). The main shortcomings of CCT lie in its lack of hierarchical structure, the unidimensional construct of the items, and the non-additivity of the rating scale data^42^. On the other hand, the Rasch analysis, which is a variant of IRT, provides a framework to model the association between the response given to the item and the underlying latent construct using an additive rating scale structure of items while enabling the PRO instrument to emulate both invariance and unidimensionality properties of the measurement. Thanks to its ability to address the significant limitations of CTT, Rasch Analysis has been used in the last decade to assess ophthalmic PROs or questionnaires^19^.

The main limitation of this study is that the shortened version of the questionnaire was not assessed in a real-world setting, and the primary goal of this study was to assess this questionnaire in a theoretical construct to assess its validity and reliability. Similar to the virtual validation proposed by Blome et al.^27^ for the Wound-QoL questionnaire, the six items of the C-OSDI-6 questionnaire were identical to the original C-OSDI-12 questionnaire. Thus, maintaining the terms of instruction for the language and response scale of the items, whereas the sequence and number of items were different. Consequently, the results of this theoretical validation can only be utilized to approximate the psychometric properties of the C-OSDI-6. In addition, the sample's mean age is 28.22 years, and the education level of the test population has to be broadened in future studies. To generalize the findings, it is necessary to carry out further research on older patients, particularly in a DED scenario that is not confined to younger contact-lens wears only. Additionally, the sample's educational level was not balanced. 98.9 percent of respondents had a tertiary degree. Inferences derived for patients with lower educational levels need to be explored and verified in future studies. A further large sample size study is necessary to strengthen the generalizability of the study results by evaluating the responsiveness of the C-OSDI-6 scores across different severities of DED.

Due to its brevity and reliability, the web-based version of C-OSDI-6 is convenient and feasible for large-scale deployment to assess dry eye symptoms. It can potentially detect ocular surface dry eye symptoms in the population as a dry eye screening tool, allowing for timely detection, treatment, and monitoring. Similar to the previous study in^20^ the assessment of the questionnaire with six items using Rasch analysis suggests that it is highly responsive and predictable. However, due to this study’s theoretical/virtual nature, further verification of its diagnostic capacity and patient acceptance needs to be explored.

## Conclusion

The C-OSDI-6 can be utilized as an easy-to-use online DED screening tool. C-OSDI-6 strongly correlates with C-OSDI-12 and good psychometrically responsiveness in Chinese dry eye disease participants. Long-term validation research on real-world clinical usage is required to corroborate the results of this virtual validation study.

## Data Availability

The datasets used and/or analyzed during the current study available from the corresponding author on reasonable request.
